# Recent advances in nanotherapeutics for the treatment of burn wounds

**DOI:** 10.1093/burnst/tkab026

**Published:** 2021-09-25

**Authors:** Rong Huang, Jun Hu, Wei Qian, Liang Chen, Dinglin Zhang

**Affiliations:** Department of Chemistry, College of Basic Medicine, Third Military Medical University (Army Medical University), Chongqing 400038, China; Department of Neurology, Southwest Hospital, Third Military Medical University (Army Medical University), Chongqing 400038, China; Institute of Burn Research, Southwest Hospital, Third Military Medical University (Army Medical University), Chongqing 400038, China; Department of plastic surgery, Southwest Hospital, Third Military Medical University (Army Medical University), Chongqing 400038, China; Department of Chemistry, College of Basic Medicine, Third Military Medical University (Army Medical University), Chongqing 400038, China; State Key Laboratory of Trauma, Burn and Combined Injury, Chongqing, 400038, China

**Keywords:** Burn wounds, Metal and metal oxide nanotherapeutics, Polymeric nanotherapeutics, Therapeutic mechanism, Wound healing

## Abstract

Moderate or severe burns are potentially devastating injuries that can even cause death, and many of them occur every year. Infection prevention, anti-inflammation, pain management and administration of growth factors play key roles in the treatment of burn wounds. Novel therapeutic strategies under development, such as nanotherapeutics, are promising prospects for burn wound treatment. Nanotherapeutics, including metallic and polymeric nanoformulations, have been extensively developed to manage various types of burns. Both human and animal studies have demonstrated that nanotherapeutics are biocompatible and effective in this application. Herein, we provide comprehensive knowledge of and an update on the progress of various nanoformulations for the treatment of burn wounds.

Highlights

- The recent progress of various nanotherapeutics for the management of burn wounds and their therapeutic mechanisms are systematically reviewed.
- Assessment of burn wounds treated with nanotherapeutics is briefly summarized.

## Background

Skin is the most active immune system organ and the largest organ of the body, as well as the main barrier between the environment and the internal organs [1]. Acute skin wounds usually result from traumas, abrasions or burns. Burn wounds are the fourth greatest cause of traumatic injuries, usually rupturing the skin layers and/or subcutaneous tissue or even damaging the viscera by physical, chemical or radioactive contact [2]. Survivors of severe burn injuries can suffer from scars, disabilities or deformities, and they can even die, which is tragic for their families and society [3].

Today, we have a deep understanding of the pathogenesis of burn wounds. The main factors determining burn wound progression include bacterial infection, excessive inflammatory reaction and low expression levels of various growth factors (GFs). Of these, bacterial infection is the most serious complicating factor [4]. In accordance with the pathogenesis of burn injuries, therapeutic strategies such as anti-infection [4], stem cell therapy [5,6] and administration of GFs to facilitate wound healing [7] have been used to treat these wounds. These interventions greatly reduce infection rate and shorten healing time [8]. Of these strategies, anti-infection plays a particularly key role in wound healing, since the injured skin is susceptible to bacteria. Antibiotics [9], metallic ions and metal oxides [10], reactive oxygen species (ROS) [11] or ROS generators [12] and other antibacterial agents have been intensely used to eradicate bacteria from the surface of burn wounds. However, transdermal or systemic administration might not ensure that adequate therapeutics reach the infection site. Furthermore, sustained antibiotic administration might increase antibiotic resistance. Another therapeutic strategy is applying GFs to the burn wound surface to shorten healing time. For example, keratinocyte formation growth factor (KGF), transforming growth factor beta (TGF-β), epidermal growth factor (EGF), nerve growth factor (NGF), basic fibroblast growth factor (bFGF), vascular endothelial growth factor (VEGF) and platelet-derived growth factor (PDGF) can trigger a cascade reaction to induce endothelial cell (EC) activation and promote neovascularization; therefore, all of these GFs have been used to treat burn wounds [13]. Nevertheless, certain physicochemical properties of GFs such as poor stabilization restrict their clinical application for this purpose.

**Figure 1.**
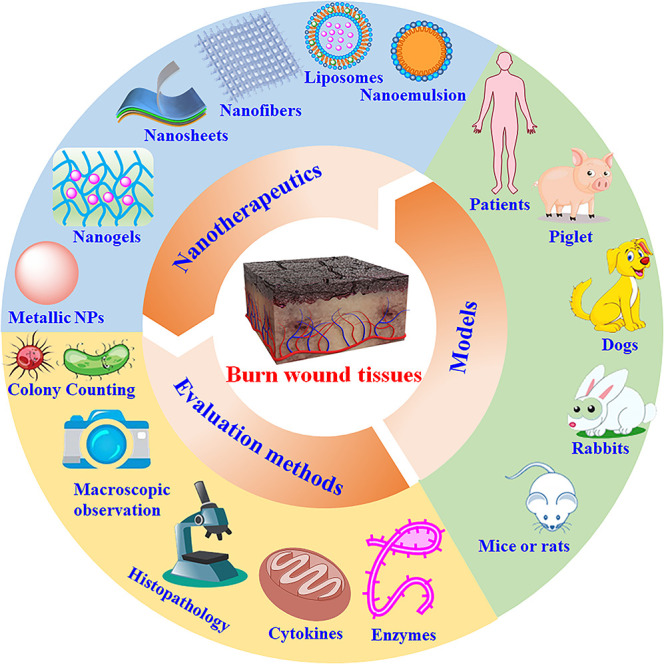
Nanotherapeutics for treatment of burn wounds. *NPs* nanoparticles

Marked breakthroughs in the field of nanotechnology offer an opportunity to solve some critical medical problems. In recent decades, nanotherapeutics and nanodiagnostics have been extensively used to diagnose and treat various diseases, including cancer [14], cardiovascular disease [15], inflammatory diseases [16], infections [17], neurological diseases [18] and dermatological diseases [19]. Compared with traditional medicine, nanotherapeutics have some treatment advantages, such as altering the physicochemical properties of conventional therapeutics, enhancing the accumulation thereof at diseased sites and decreasing drug dosage and dose frequency [20]. The great recent progress in nanomedicine also provides a chance to develop nanotherapeutics that efficiently prevent infection and facilitate healing of burn wounds (Figure 1). Some nanomaterials (e.g. silver [Ag], zinc oxide [ZnO] nanoemulsions and chitosan nanoparticles [NPs]) can serve as anti-bacterial agents to prevent infection of burn wounds, since these materials have intrinsic anti-bacterial efficacy [10]. In addition, researches have encapsulated antibiotics in polymeric materials *(*e.g*.* cellulose, polysaccharide) to treat burn wounds [21,22]. GFs have been encapsulated into NPs to boost cell proliferation, which helps facilitate wound healing [23,24]. Nanotherapeutics for the treatment of burn wounds have some outstanding advantages, such as broad-spectrum anti-bacterial efficacy, overcoming bacterial drug-resistance, shortening wound healing time and satisfactory biocompatibility. The efficacy and biocompatibility of nanotherapeutics for the treatment of burn wounds have both been demonstrated in human and animal models. Although nanotherapeutics have made great achievements in treating these wounds, progress in this field continues, as summarized by several reviews [23,25]. Herein, we provide a comprehensive acknowledgment of and an update on the progress of recent research into various nanoformulations for treatment of burn wounds.

## Review

### Strategies for burn wound treatment

**Figure 2.**
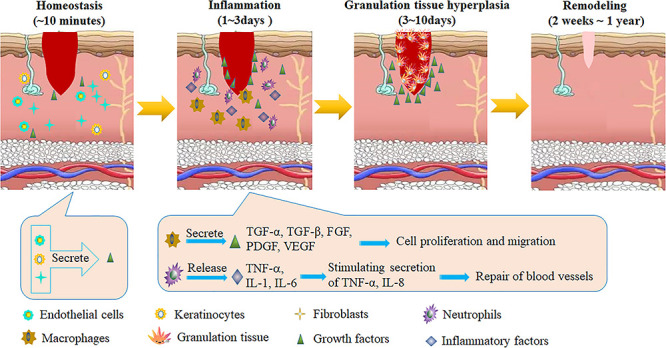
The healing mechanisms of burn wounds. *TGF-α* transforming growth factor alpha, *FGF* fibroblast growth factor, *PDGF* platelet-derived growth factor, *VEGF* vascular endothelial growth factor, *IL-8* interleukin 8, *TNF-α* tumor necrosis factor alpha

**The mechanism of burn wound healing** Wang, Jahromi *et al.* have systematically reviewed the mechanism of burn wound healing [7,23]. The process usually includes four phases: homeostasis, inflammation, granulation tissue hyperplasia and re-epithelialization/remodeling (Figure 2) [7]. Homeostasis occurs in the early stages of burn wounds (within 10 min after wound infliction) to minimize damage (Figure 2a). In this phase, a blood clot containing hyaline, fibronectin (FN), fibrin and thrombin-sensitive protein (TSP) forms a scaffold-like matrix for the migration of fibroblasts, leukocytes, keratinocytes and ECs as well as for the resulting aggregation of GFs at the wound site [26]. An inflammatory reaction appears 1–3 days after burn wounds. In this phase, neutrophils are accumulated at the burn sites which release inflammatory factors such as tumor necrosis factor alpha (TNF-α) and interleukins-1 and -6 (IL-1, IL-6); this activates the inflammatory response and stimulates VEGF and IL-8 secretion in order to repair blood vessels (Figure 2b). In addition, monocytes are transformed into activated macrophages that accumulate at the wound site to produce various GFs such as TGF-α, TGF-β, FGF, PDGF and VEGF to stimulate cell proliferation and migration [27]. The granulation tissue’s hyperplasia phase, which includes re-epithelialization, neovascularization and granulation tissue formation, usually occurs 3–10 days after the burn wound is inflicted (Figure 2c). In this phase, re-epithelialization induced by activated cytokines causes expansion of keratinocytes, ECs, stem cells and fibroblasts at the wound site. In addition, high expression of various GFs (*e.g.* VEGF, PDGF, FGF-β and GM-CSF) at the site can facilitate growth of ECs. Alternatively, fibroblasts, granulocytes and macrophages can form granulation tissue that becomes fibrous tissue, eventually forming a scar [28]. In the 2–3 weeks to 1 year after the burn occurs, the wound heals completely, this is defined as the tissue plasticity period (Figure 2d) [29].

**Strategies for accelerating burn wound healing** Clinical therapy for burn wounds usually includes four procedures: preventing further injury to the wound, wound cleaning and drainage, prevention of wound infection and promotion of wound healing. Therapeutic strategies for the various treatment principles are summarized in Table 1.

**Table 1:** Current strategies for accelerating burn wounds healing

**Principles**	**Strategies**	**Applied drugs or materials**
Proper first aid	Preventing further injury, immediate cold treatment, promote microcirculation of wounds, keeping wound in a wet environment, providing an ideal wet microenvironment for wounds, anti-inflammatory and antioxidant therapy	Alprostadil, Chinese herbs (shengmai, safflor yellow, etc.) and vitamin C
Early debridement and complete drainage	Removal of the necrotic tissue and foreign matter in the wound, and drainage of blister fluid or other wound effusion, to provide a clean environment for wounds	Ultrasound, proteases, maggot, etc.
Prevention and treatment of wound infection	Topical and systemic application of antimicrobial agents	Chinese herbs (Coptis chinensis, phellodendron, eucalyptus leaves, etc.), chemical disinfectant (iodophor, hydrogen peroxide, chlorhexidine acetate, benzalkonium bromide, etc.), antimicrobial agents (mupirocin, fusidic acid, silver sulfadiazine, mafenide, etc.)
Promoting wound healing	Addition of growth factors	FGF, EGF, PDGF, GM-CSF, etc.
	Application of functional dressings	Hydrocolloids, hydrogels, alginates, foams, hydrofibres, anti-microbial dressings, etc.
	Negative pressure wound therapy	Negative pressure pump, sealing film, negative pressure patch or biocompatible porous materials
	Platelet-rich plasma therapy	PDGF, TGF-β, IGF, EGF, VEGF

*FGF* fibroblast growth factor, *EGF* epidermal growth factor, *PDGF* platelet-derived growth factor, *GM-CSF* granulocyte macrophage colony stimulating factor, *TGF-β* transforming growth factor β, *IGF* insulin-like growth factor, *VEGF* vascular endothelial growth factor

Debridement and drainage comprise the first step to keeping the wound clean and decreasing the chance it will become infected. After debridement and drainage, infection prevention must be considered since bacterial infection significantly affects the wound healing process. Serious infection can damage the remaining epithelial tissue, which prolongs wound healing time. Importantly, if sepsis occurs after wound infection, epithelial growth is terminated, which makes healing difficult [30]. In addition, partial infection occurring on the wound surface will lead to persistent inflammatory response, resulting in an increase of necrotic tissue, blockage of collagen formation, prevention of tissue regeneration and prolongation of the recovery stage. Consequently, preventing infection of the wound is vital to facilitate its healing. Susceptible bacteria groups such as Methicillin-resistant *Staphylococcus aureus* (*S. aureus*) (MRSA), *Pseudomonas aeruginosa* (*P. aeruginosa*) and *Escherichia coli* (*E. coli*) are the predominant pathogens that delay wound healing. Currently, therapeutics used for infected wounds include immune-based antibacterial agents (such as antimicrobial peptides) [31], therapeutic micro-organisms [32], various antibiotics and ROS [33]. Antibiotics are the most effective therapy for wound infections. In addition, many metallic ions or particles such as gold (Au), silver (Ag), zinc (Zn) and copper (Cu) show broad-spectrum antibacterial activity and have been widely used to treat various wound infections.

In addition to controlling wound infection, appropriate control of inflammatory reaction at the wound site is beneficial for wound healing, as is the administration of GFs such as GM-CSF, TGF, VEGF, bFGF and PDGF [34]. In conclusion, thanks to in-depth research into the pathological mechanisms of burn wounds, various therapeutic strategies have been developed to treat these wounds [3], significantly reducing risk of infection and obviously shortening healing time.

**Table 2:** Metal and metal oxide nanotherapeutics for treatment of burn wounds

**Nanomaterials**	**Size**	***In vitro* assays**	**Animal models**	**Reference**
Ag NPs	-	*P. aeruginosa*	Rats bearing burn wound infected with *P. aeruginosa*	[52]
	15 nm	*-*	Rats bearing burn wound	[122]
	7–26 nm	*S. aureus*	Mice bearing acute burn wound infected with *S. aureus*	[71]
	82–140 nm	*E. coli, P. aeruginosa, S. aureus*	Promoting healing of burn wound on rats	[123]
	-	-	Patients with 15–40% partial thickness thermal burns	[55]
AgCl NPs	42 ± 15 nm	-	Rats with second-degree burn wound	[120]
AgSD NPs	~282 nm	*S. aureus, P. aeruginosa, E. coli*	Rats with scald wound	[124]
Ag/AgCl NPs	~10 nm	*S. aureus, E. coli*	Mice bearing second degree burn wound	[54]
Au NPs	520–525 nm	-	Repair of burn wound in rats	[68]
	28–37 nm	*S. aureus*	Mice bearing burn wound infected with *S. aureus*	[69]
	25 nm	*-*	Improving mitochondrial activity of rats bearing burn wound	[70]
	~10 nm	*P. aeruginosa*	Against infection of rats bearing burn wound	[125]
ZnO NPs	30–80 nm	*E. coli, P. vulgaris, S. aureus*	Acceleration healing of wound on rats	[66]
	~100 nm	*E. coli, S. aureus*	Rats with burn wound	[65]

*S. aureus* staphylococcus aureus, *P. aeruginosa* pseudomonas aeruginosa, *E. Coli* escherichia coli, *NPs* nanoparticles

**Nanotherapeutics for promoting burn wound healing** The rapid development of nanotechnology over the past 20 years has provided opportunities for the treatment of various diseases. Nanotherapeutics are the drugs, biomacromolecules (e.g. DNA, peptides, proteins) and therapeutic materials (e.g. some metals/metal oxides, chitosan), or pharmaceuticals that have nanoscale structure in at least one dimension [35]. Nanotherapeutics have multiple advantages in treating bacterial infection, as they can (1) enhance interactions between drugs and bacteria or change the pathway of the drug to improve its anti-bacterial effects; (2) increase drug concentration at infection sites, which helps reduce drug dosage and alleviate toxic side effects; (3) improve drug penetration into tissue barriers and bacterial biofilms to overcome bacterial resistance; and (4) improve the stability and prolong the half-life of drugs [36]. Due to their abovementioned advantages, polymeric, metal, metal oxide and other nanotherapeutics have been widely employed to treat burn wounds. Metallic nanomaterials (Ag, ZnO, Au, Cu) have broad-spectrum anti-bacterial activity by breaking down biofilms, damaging bacterial DNA or generating ROS to inhibit bacterial growth [37,38]. However, the toxicity of these nanotherapeutics should be considered, as it can restrict their further *in vivo* application. Compared with metal nanomaterials, polymeric nanomaterials (e.g. polysaccharide, polyester, polyamide) have excellent biocompatibility and biodegradability and have been extensively used in various biomedical fields. Some cationic polymeric nanomaterials, such as chitosan, have bactericidal and bacteriological properties due to the positive charge of the polymer; they adhere to bacterial surfaces, inducing damage of the membrane wall, which prevents microbial growth [39]. Encapsulating antibiotics in polymeric nanomaterials is another crucial strategy in preventing wound infection [40], while encapsulating GFs in such materials to shorten wound healing time has also been extensively investigated [41]. In summary, nanotherapeutics have been developed to treat burn wounds and exhibit good antibacterial effect, shortened wound healing time and reduced bacterial drug resistance. Therefore, they are a promising prospect for clinical treatment of burns [23]. In the following section, we summarize the uses of various nanomaterials and nanotherapeutics in burn wounds and discuss their merits and disadvantages in such applications.

### Metal and metal oxide nanotherapeutics for burn wound healing

Metal and metal oxide nanotherapeutics (e.g. Au, Ag and ZnO NPs) have been broadly employed to treat burn wounds as well as various other cutaneous infections [42,43], since they possess a broad spectrum of antimicrobial properties. Furthermore, these nanotherapeutics can overcome bacterial resistance in multiple ways, such as DNA damage, enzyme activity disruption, cell wall destruction, plasmid damage, inhibition of biofilm formation and oxidative stress [36,44]. The applications of metal and metal oxide nanotherapeutics for the management of burn wounds are summarized in Table 2.

**Ag NPs** Ag ions are exceptional anti-microbial agents due to their superior antibacterial capability and broad-spectrum antimicrobial effects against bacteria, viruses and other eukaryotic micro-organisms [45,46]. Ag NPs in particular have better antimicrobial activity than ionic silver due to their superior permeation and retention effects [47]. Therefore, they have been extensively used to treat burn wounds [48–50]. For example, ‘Acticoat’, an Ag NPs-based bandage for treating burn wounds, has been approved by the US Food and Drug Administration (FDA) and is used in clinical practice. Ag NPs-based topical creams, ointments and gels have been widely used to prevent the spread of microbial infections in injured patients [51].

For stabilization and convenient administration, Ag NPs are usually loaded on polymer, inorganic materials or animal tissues to prepare antibacterial materials for the treatment of burn wounds. Susceptible pathogenic bacteria such as *P. aeruginosa, S. aureus* and *E. coli* have been used to evaluate the *in vitro* antibacterial activity of Ag NPs; meanwhile, to assess these NPs’ *in vivo* efficacies, rats and mice with bacterially infected burn wounds have been used as animal models. For example, porcine-derived small intestinal submucosa (PSIS) is an acellular, xenogenic biological material widely used to repair and regenerate wounded and dysfunctional tissues. Zhang *et al.* used Ag NPs-loaded PSIS as a biological-derivative dressing for treatment of *P. aeruginosa*-infected partial-thickness burn wounds in a rat model [52]. The authors found that Ag NPs-loaded PSIS can significantly promote wound healing and recover the normal growth of rats due to suppression of inflammation and stimulation of re-epithelialization during the wound healing process. Histological section results reveal that Ag NPs-loaded PSIS can obviously decrease inflammatory cell infiltration and accelerate re-epithelialization and neovascularization of burned tissues.

Release of Ag^+^ from Ag NPs often causes side effects due to the toxicity of Ag^+^ in mammalian cells, resulting in argyria and argyrosis in humans [53]. To circumvent this issue, a highly efficient, stable and biocompatible Ag NPs-based bactericide was developed via fabrication of ultrafine Ag/AgCl NPs coated with graphene; these have been successfully used to treat burn wounds in animal models [54]. An Ag/AgCl nanophotocatalyst with negligible release of Ag^+^ can generate a high number of oxidative radicals to kill bacteria. Histopathological results show that the Ag NPs-loaded graphene can obviously promote epidermal regeneration, which is beneficial in accelerating burn wound healing.

**Figure 3.**
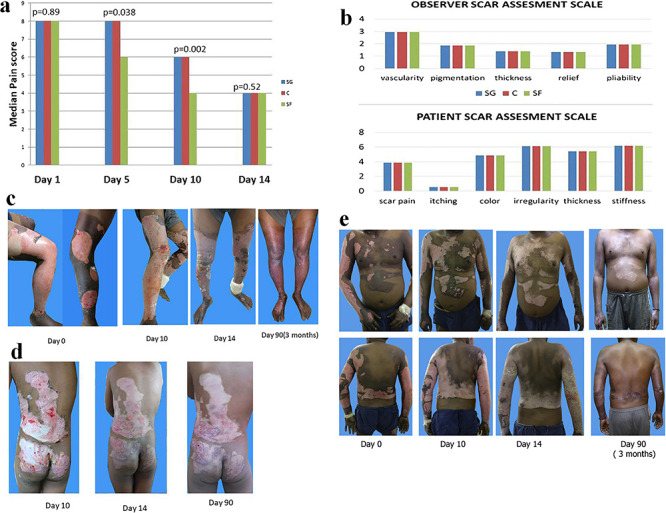
Ag-based nanotherapeutics for treatment of burn wound on patients with 15-40% partial thickness thermal burns. (**a**) Comparison of assessment of pain; (**b**) assessment of scar quality at 3 months [silver nanoparticle gel (SG), nanosilver foam (SF), collagen (C)]; (**c–e**) clinical photographic assessment in patient; (**c**) SG: left leg, SF: right leg, C: bilateral thighs; (**d**) SG: left buttock, SF: right buttock, C: back torso; (**e**), SG: left upper limb, C: right upper limb, SF: torso [55]. (Copyright 2018 by Elsevier Ltd)

Importantly, the therapeutic efficacy of Ag NPs for treatment of burn wounds has been evaluated in patients with 15–40% partial thickness thermal burns. For instance, Gaba and co-workers compared the efficacy of silver nanoparticle gel (SG), nanosilver foam (SF) and collagen (C) dressings in partial-thickness burn wounds [55]. Interestingly, pain scores were significantly decreased when patients were treated with SF dressing at days 5 and 10 (Figure 3a), indicating that this treatment could relieve patient pain during therapy. Scar quality at 3 months as assessed by observers and patients was found to be similar across various parameters (Figure 3b). In particular, clinical-assessment results suggested that SF dressings were more efficacious for re-epithelialization and healing than either SG or C dressings in partial-thickness burns (Figure 3c–e) [55]. These results indicate that Ag nanotherapeutics show promising potential in clinical practice for the treatment of burn wounds.

**ZnO NPs** Zinc, which has a long lifetime in living cells, is an essential micronutrient in tissue regeneration and can increase keratinocyte count to accelerate wound healing [56–60]. The underlying antibacterial mechanism is that ZnO NPs are captured by the bacterial cell wall, resulting in ineffectiveness and cleavage of the cell membrane of bacteria [61,62]. Importantly, ZnO NPs can improve cell adhesion, proliferation and cell migration via GF-mediated pathways due to Zn’s semiconductor properties. Therefore, ZnO NPs can also serve as sustained sources of ionic Zn for wound treatment due to their anti-bacterial, anti-inflammatory and low cytotoxicity properties [63,64], and indeed they have been widely used to treat various types of burn wounds.

ZnO NPs are usually loaded on polymer or polysaccharide for bandage preparation, which makes administration convenient. ZnO NPs-loaded bandages have been extensively developed for various types of burn wounds in animal models. For example, a ZnO NPs-loaded keratin-chitosan bandage displayed high porosity, which was encouraging for the augmentation of fibroblasts [65]. This bandage demonstrated good antibacterial activity, tensile strength and biodegradation. *In vivo* experiments demonstrated that this bandage could facilitate wound curing via quicker skin cell construction and collagen development. Alternatively, plant extracts such as *Barleria gibsoni* have also been used to stabilize ZnO NPs; these NPs also showed good antibacterial properties and were proven to be efficient antimicrobial formulations for healing burn infections in rats [66].

**Au NPs** Au NPs possess robust physical and chemical stability in biological media, as well as superb biocompatibility. They have been proven capable of penetrating the stratum corneum and thus the skin barrier [67]. For example, phytochemical-capped Au NPs have been used for transdermal treatment of skin with surgical or burn wounds [68]. One study investigated the biological activities and therapeutic potential of phytochemical-capped Au NPs by using them to treat the dorsal skin of rats via transdermal drug delivery in order to regenerate surgically wounded and burnt skin. *In vivo* experiments demonstrated that the treatment effected by Au NPs in these rats accelerated the growth efficiency of dorsal skin, increased dermal and epidermis thickness, suppressed collagenase expression and contributed to the induction of antioxidants. Au NPs have also been encapsulated in Pluronic 127 and hydroxypropyl methylcellulose to prepare thermoresponsive gels for treating infected burn wounds in mice [69]. Histopathological experiments have demonstrated that these Au NPs formulations show antibacterial activity with the highest wound healing values. In addition, Au NPs can significantly reduce oxidative damage parameters and obviously increase levels of antioxidant defense enzymes in burn wound tissues; this indicates that they can improve mitochondrial functioning and oxidative stress parameters, which contribute to tissue repair [70].

In conclusion, metal and metal oxide nanotherapeutics have been extensively employed to facilitate burn wound healing due to their strong broad-spectrum antibacterial properties. *In vivo* experiments have verified that these nanotherapeutics can distinctly decrease bacteria counts in burnt tissues [52,69,71]. Importantly, the therapeutic efficacy of Ag NPs has been evaluated in patients with satisfactory outcomes [55]. Metal and metal oxide nanotherapeutics therefore show promising potential in clinical practice for the treatment of various types of burn wounds.

### Polymeric nanotherapeutics for management of burn wounds

As previously mentioned, polymeric nanomaterials, with their excellent biocompatibility and biodegradation, have been widely employed to fabricate various nanotherapeutics (NPs, nanoemulsions, nanogels, liposomes, nanofibres and nanosheets) for the treatment of burn wounds [25]. Either hydrophilic/hydrophobic drugs or GFs can be encapsulated into polymeric nanomaterials to form various nanotherapeutics [72]. Polysaccharides (e.g. chitosan, dextran), polyesters (e.g. PLGA), phospholipids, hyaluronic acid (HA) or polyvinyl alcohol can serve as carriers for encapsulating therapeutics [73,74]. The application of various polymeric nanotherapeutics for the treatment of burns is summarized in Table 3.

**Table 3:** Polymeric nanotherapeutics for treatment of burn wounds

**Nanoformulations**	**Carriers**	**Payloads**	**Size**	***In vitro* assays**	**Animal models**	**Reference**
Nanogels	*Aerva javanica*	Ag NPs	8–21 nm	*P. aeruginosa,* MRSA	Mice with burn wound infected with *P. aeruginosa* or MRSA	[82]
	Sodium-alginate	Ag NPs	-	*E. coli, S. aureus*	Mice bearing burn wound	[83]
	*Aloe vera* gel/carbopol 940	Ag NPs	-	-	Rats bearing second degree burn wound	[84]
	Gelatin/HA/chitoson	ZnO/CuO	-	Fibroblast cells	Rats bearing second degree burn wound	[85]
	Gelatin/pluronic	Curcumin	7–16 nm	Fibroblast cells	Mice bearing second degree burn wound	[86]
	Pluronic/chitosan	EGF	-	Human keratinocytes	Mice bearing second degree burn wound	[87]
	Polystyrene	Peptide	-	-	Rats bearing burn wound	[88]
	PEG	-	-	NIH3T3 cells	Animal with second degree burn wound infected with *P. aeruginosa*	[126]
	Silk fibroin-sodium alginate/poly(*N*-isopropylacrylamide)	Vancomycin/EGF	-	Fibroblast cells	*S. aureus* infected rats bearing burn wound	[127]
Nanofibres	Gelatin/poly-3-hydroxybutyric acid	AgSD NPs	100–140 nm	-	Rats bearing pseudomonas infected burn wound	[89]
	Zn–Al layered double hydroxides/PVA	Cefotaxime	-	-	Rats bearing burn wound	[90]
	Alginate	Lavender oil	91–93 nm	*S. aureus*	Promoting healing of burn wound on mice	[91]
	poly(octyl cyanoacrylate)	Fumarate	800 nm	*-*	Recovery of mild skin burn on mice	[92]
	Gelatin	Dopamine/antibiotics	~ 1000 nm	*Candida albicans, S. aureus, E. coli, P. aeruginosa, et al.*	Treatment of burn injury on piglets	[93]
	Gelatin/starch	*Lawsonia inermis*	87 nm	*S. aureus, E. coli*	Antibacterial and anti-inflammatory for burn wound on mice	[94]
	-	Peptide amphiphile	-	hFBs, HUVECs	Enhancing burn wound healing on rats	[95]
	-	Peptide	-	-	Rat bearing burn wound for investigation of hair growth	[96]
	Heparin mimetic peptide	-	-	-	Promoting regeneration of burn injury on mice	[97]
	PCL/chitosan/PVA	-	-	-	Rats bearing full thickness round burn wound	[128]
	PCL/chitosan/PVA		~136 nm.	-	Dogs bearing full-thickness third-degree burn wounds	[121]
Nanosheets	Bacterial cellulose	ZnO NPs	-	*E. coli, P. aeruginosa, S. aureus, Citrobacter freundii*	Treatment of burn wound on mice	[10]
	PLA/PVA	AgSD NPs	38 nm	*MRSA*	Mice bearing burn wound infected with MRSA	[98]
	Chitosan	Ag NPs	7–33 nm	*S. aureus, P. aeruginosa*	Antibacterial/tissue regeneration of burn wound on rats	[99]
	-	Ag NPs	-	*-*	Rabbits with deep second-degree scald models	[100]
	Chitosan/sodium alginate	Tetracycline	142–177 nm	*P. aeruginosa*	Mice bearing burn wound infected with *P. aeruginosa*	[129]
	Chitosan/dextran	siRNA	-	NIH-3 T3, HeLa, MDA-MB-231 cells	Reduction of cutaneous scar contraction in third-degree burn on rats	[101]
	PEO	Silk fibroin	-	-	Acceleration of burn wound healing on rats	[102]
	PLGA/chitosan	Minocycline	-	*S. aureus, P. aeruginosa*	Acceleration of burn wound healing on rats	[130]
Nanoemulsions	Histidine	Ag NPs	120 nm	*Klebsiella pneumoniae*	Mice bearing third-degree burn wound infected with *K. pneumoniae*	[131]
	Labrasol®, Plurol®	AgSD	25–71 nm	*E. coli, S. aureus*	Treatment of burn wound on mice	[132]
	Virgin coconut oil, olive oil, vitamin E	Bromelain	27–126 nm	-	Treatment of thermal-induced burn wound on rabbits	[112]
	Tween 80, poloxamer 147, lutrol F68, span 40	Fusidic acid	20–110 nm	*S. aureus*	Mice bearing burn wound infected with *S. aureus*	[109]
	Tween 80/PEG	Chlorhexidine acetate	~63 nm	*S. aureus*	MRSA-infected burn wound mice	[108]
	Poly-3-caprolactone-pluronic	Chloramphenicol/essential oil	123 nm	*S. aureus, P. aeruginosa, C. albicans, Candida glabrata*	Treatment of MRSA–candida co-infected chronic burn wound on mice	[133]
	Vegetable oil	-	-	*P. aeruginosa*	Reduction of bacterial wound infection and inflammation after burn injury on rats	[107]
	BAC/CPC/poloxamer 407/tween 20	-	212–336 nm	-	Rats with scald burn infected with *P. aeruginosa or S. aureus*	[110]
Liposomes	DMPC/CTAB	Chlorine e6	~ 110 nm	*C. albicans*	Rats bearing skin burn wound infected with *C. albicans*	[134]
	Lecithin/cholesterol	bFGF	~ 100 nm	NIH/3 T3 fibroblast cells	Mice bearing deep second-degree scald	[135]
	DOTAP/tween 80	EGF	16–87 nm	HaCaT	Rats bearing burn wound	[114]
	-	Keratinocyte growth factor	-	-	Improving wound healing of scald burn on mice	[136]
Lipid NPs	Poloxamer®F-127, gellucire® 44/14	Fusidic acid	~ 310 nm	MRSA	Prevention of infection from burn wound on mice	[116]
Dendrimers	Dendrimer	Ag NPs	-	RAW264.7, J774.1 cells	Anti-inflammatory for mice bearing burn wound	[118]
MSCs	-	Fe_3_O_4_/PDA NPs	-	MSC	Rats bearing laser burn wound	[117]
NPs	PCL	TiO_2_–Ag	~16 μm	*E. coli and S. aureus*	Mice bearing burn wound	[119]

*S. aureus* staphylococcus aureus, *P. aeruginosa* pseudomonas aeruginosa, *E. Coli* escherichia coli, *C. albicans* candida albicans, *MRSA* methicillin-resistant Staphylococcus aureus, *NPs* nanoparticles, *EGF* epidermal growth factor, *hFBs* normal human fibroblast, *PEG* polyethylene glycol, *HUVECs* human umbilical vein endothelial cells, *PCL* poly(caprolactone), *PVA* poly(vinyl alcohol), *PEO* polyethylene oxide, *BAC* benzalkonium chloride, *CPC* cetylpyridinium chloride, *DMPC* dimyristoyl-sn-glycero-phosphatidylcholine, *CTAB* cetyltrimethyl ammonium bromide, *DPTAP* 1,2-Dioleoyl-3-trimethylamonium propane chloride, *bFGF* basic fibroblast growth factor, *MSC* mesenchymal stem cell

**Nanogels** Nanogels, which hare particle size <200 nm, are composed of hydrophilic or amphiphilic polymers through physical or chemical crosslinking with nanoparticles [75]. Polymeric materials such as polyacrylic acid [76], polyacrylamide [77], Pluronic [78], polysaccharide [79], polyethylene glycol and derivatives thereof are usually employed to prepare nanogels. These materials can be crosslinked through amine reactions, click chemistry, photo-induced crosslinking, physical crosslinking or heterogeneous polymerization of monomers to form nanogel systems. Hydrogel sheet or plasters, impregnated hydrogels and amorphous hydrogels have been commercialized for the treatment of burns and other skin wounds [80,81].

Metal or metal oxide antimicrobial agents (e.g. Ag or ZnO NPs) are loaded on nanogels to prevent infection and facilitate healing of burn wounds [82–85]. *In vitro* experiments indicate that these nanogels loaded with metallic antimicrobial agents have excellent antibacterial activity against Gram-negative and -positive bacteria such as *P. aeruginosa, E. coli, S. aureus* and MRSA. Their therapeutic efficacy has also been evaluated in animal models. *In vivo* experiments have demonstrated that nanogels loaded with metallic antimicrobial agents can significantly facilitate wound healing by reducing inflammation, eliminating pathogenic bacteria and accelerating tissue regeneration. Histological analysis of wounds has verified that nanogel treatment can promote granulation tissue formation, collagen deposition, neovascularization and re-epithelialization, all of which are beneficial in facilitating wound healing.

In addition to metallic antimicrobial agents, phytomedicines (e.g. curcumin), peptides and GFs (e.g. EGF) have also been encapsulated into nanogels for treatment of burn wounds [86–88]. For example, Dang *et al.* fabricated injectable nanocurcumin-dispersed gelatin/Pluronic nanogels for this purpose [86]. The nanocurcumin-dispersed gelatin/Pluronic solution can form nanogels on warming, up to 35°C. Curcumin-loaded nanogels have good biocompatibility and can promote fibroblastic proliferation. *In vivo* experimental results suggested that the application of curcumin-loaded nanogels can accelerate the wound healing process.

In conclusion, nanogels as dressings possess multiple advantages such as good biocompatibility and biodegradation and easy preparation. Importantly, such dressings can provide a moist, anti-infectious healing environment and can be easily removed without trauma. Consequently, various nanogels containing antibacterial agents or GFs have been successfully used to treat burn wounds.

**Figure 4.**
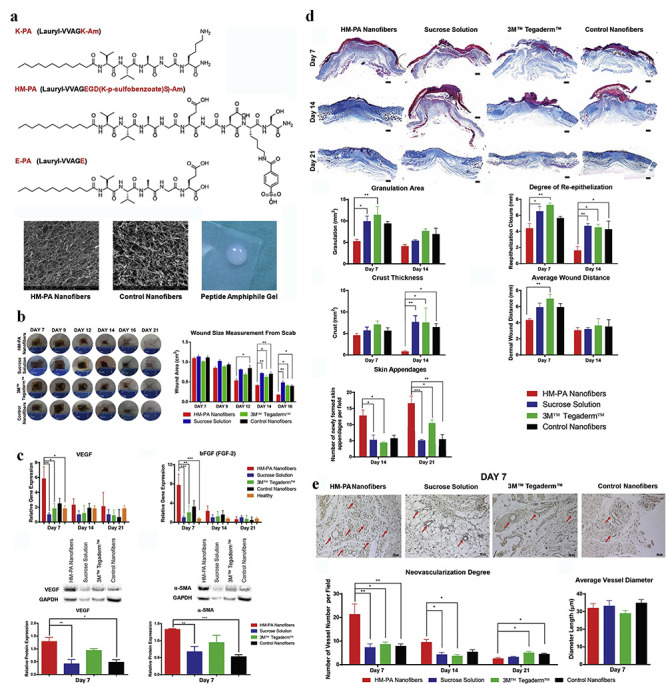
Nanofibres for management of burn wounds. (**a**) Chemical structures of peptide and characterization of peptide nanofibres at pH 7.4 by SEM. (**b**) Representative images of burn wounds after nanofibres treatment and quantification of wound areas treated with HM-PA peptide nanofibres. (**c**) Protein and mRNA levels of genes associated with angiogenesis and wound repair at the burn wound sites; qRT-PCR analyses were performed for vascular endothelial growth factor (VEGF) and basic fibroblast growth factor (bFGF), while Western blot analyses were performed for VEGF and α-smooth muscle actin (alpha-SMA). (**d**) Masson’s trichrome staining of wound tissues and quantitative analysis of granulation tissue, re-epithelization, crust area, wound distance and skin appendages of burn wounds. (**e**) Staining of blood vessels and quantification of blood vessels. ^*^p < 0.05, ^**^p < 0.01, and ^***^p < 0.001. *K-PA* positively charged peptide amphiphile, *E-PA* negatively-charged peptide amphiphile, *SEM* scanning electron microscope, *HM-PA nanofibres*, heparin-mimetic peptide nanofibres [97]. (Copyright 2017 by Elsevier Ltd)

**Nanofibres** Nanofibres are fibres 1–100 nm in diameter. Polymers such as polyurethane, polydimethylsiloxane, polyethylene terephthalate, polyethersulfone, poly (acrylic acid) (PLA) and poly (methyl methacrylate) have been employed to fabricate nanofibres. Antimicrobials (e.g. Ag NPs, cefotaxime), plant extracts (e.g. *Lawsonia inermis*, lavender oil) and peptides are loaded onto nanofibres to treat bacteria-infected burn wounds and facilitate the healing thereof [89–97]. For instance, heparin mimetic-peptide nanofibres have been employed to promote regeneration of full-thickness burn injuries in order to alleviate the progressive loss of tissue function at the post-burn wound site (Figure 4a, b) [97]. Interestingly, bioactive nanofibres can form scaffolds that recapitulate the structure and function of the native extracellular matrix (ECM) by signaling peptide epitopes, which can trigger angiogenesis via their affinity for GFs. *In vivo* animal experiments indicate that heparin-mimetic peptide nanofibres can support the repair of full-thickness burn injuries by mediating wound contraction and re-epithelialization, preventing scar formation and stimulating the development of skin appendages (Figure 4d). Investigation into the underlying mechanism has shown that peptide nanofibres can promote GF (e.g. VEGF, bFGF) overexpression and facilitate neovascularization at burn wound sites (Figure 4c, e).

Self-assembling short-peptide nanofibres have been developed to boost aesthetic repair of burn wounds [96]. Hair follicle growth, hair growth length, and expression of bFGF and EGF were evaluated in a rat model treated with nanofibres. The *in vivo* animal experiments indicated that levels of all of the above parameters in the experimental group were better than those in the control group. These results suggest that self-assembling short-peptide nanofibres might potentially facilitate the aesthetic repair of burn wounds.

In summary, because nanofibres can simulate the fibrous component of natural ECM, they can therefore serve as ECM analogues for skin regeneration. In addition, nanofibres can form a protective barrier against penetration of pathogens into wounds, retain moisture in damaged skin, allow for gas exchange and absorb wound exudate. In addition, nanofibres can also be loaded with various bioactive molecules, such as growth and angiogenic factors, to facilitate healing of burn wounds.

**Figure 5.**
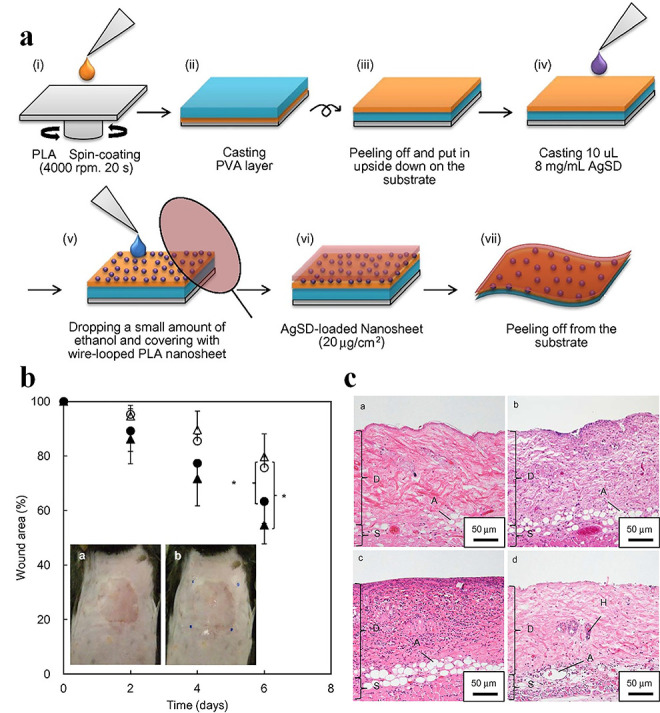
Silver sulfadiazine (AgSD) nanoparticles (NPs)-loaded nanosheets for treatment of burn wound in mice. (**a**) Scheme for the preparation of AgSD-loaded nanosheets. (**b**) Wound healing potency of AgSD-loaded nanosheets 6 days after treatment ((● no infection, ο sham, ∆ AgSD (−), ▲ AgSD (+)), (*n* = 6, *p < 0.05) (inset) (a and b) macroscopic images of the wound before a and after b applying the AgSD-loaded nanosheets. (**c**) Histological images of the wound area 3 days after injury, a no infection, b sham, c AgSD (−), and d AgSD (+). *D* dermis, *S* subcutaneous layer, *A* adipose tissue, *H* hair root, *PLA* poly(lactic acid), *PVA*, poly(vinyl alcohol) [98]. (Copyright 2015 by Elsevier Ltd)

**Figure 6.**
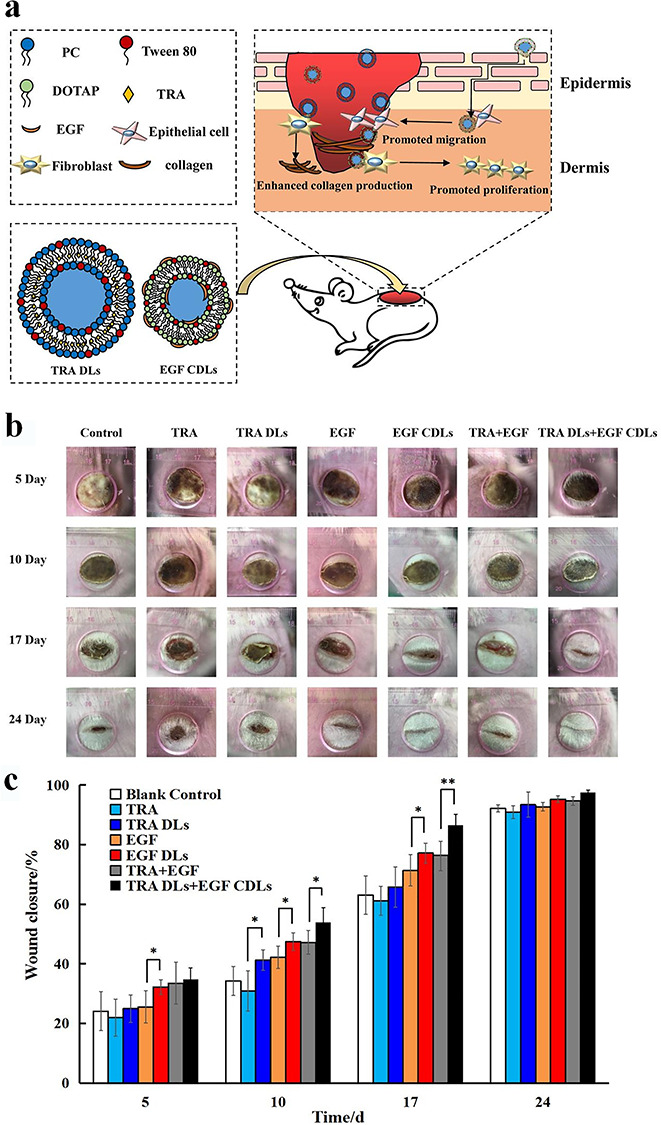
EGF-loaded liposomes for treatment of burn wound in rats. (**a**) Graphic illustration. (**b**) Representative photos of full partial-thickness burn wound treated with EGF-loaded liposomes at various time points. (**c**) Wound closure rate of full partial-thickness burn wound at various time points. ^*^p < 0.05, ^**^p < 0.01. *TRA* all-*trans* retinoic acid, *TRA DLs* all-*trans* retinoic acid loaded deformable liposomes, *EGF CDLs* epidermal growth factor cationic deformable liposomes, *DOTAP* 1,2-dioleoyl-3-trimethylamonium propane chloride, *PC* phosphatidyl choline [114]. (Copyright 2019 by Royal Society of Chemistry)

**Nanosheets** Nanosheets, which are tens of nanometers thick, have unique physical properties such as high flexibility, strong adhesiveness and high transparency [98]. Wounds coated with nanosheets not only protect wounds from the environment but also provide a visual field for the observation of wound recovery. Consequently, bacterial-cellulose and polymer (e.g. PLA, chitosan, polyethylene oxide) nanosheets loaded with antimicrobial agents (e.g. ZnO NPs, Ag NPs) [10,98–100], short interfering ribonucleic acid (siRNA) [101], and silk fibroin [102] have been extensively used to treat various types of burn wounds. For example, Ito *et al.* prepared silver sulfadiazine (AgSD)-loaded PLA nanosheets and tested their antimicrobial properties (Figure 5a) [98]. These nanosheets had a high degrees of flexibility, adhesive strength and transparency that made them suitable for treating burn wounds. An *in vitro* Kirby–Bauer test indicated that these nanosheets exerted antimicrobial efficacy against MRSA. *In vivo* evaluation using a mouse model of infected partial-thickness burn wounds verified that the nanosheets significantly reduced MRSA bacteria count on the lesions and suppressed the inflammatory reaction (Figure 5b, c). These drug-loaded transferrable nanosheets have high potential for treating burn wounds via controlled drug-release. Nanosheets have also been employed to deliver siRNAs for local silencing of GFs to reduce cutaneous scars. For instance, connective-tissue growth factor (CTGF) has been demonstrated to function as a key mediator of scar formation *in vivo*, and mediating its expression is an effective way to reduce scar formation [101]. Castleberry *et al.* exploited nanosheets for controlled delivery of siRNAs to improve scar outcomes in a third-degree burn-induced scar model in rats [101]. The authors demonstrated that knockdown of CTGF can significantly alter local expression of alpha-smooth muscle actin (α-SMA), tissue inhibitor of metalloproteinase-1 (TIMP-1) and collagen (Col1a1), which play roles in scar formation. The authors also verified that improved tissue remodeling, reduced scar contraction and regeneration of papillary structures within the healing tissue occurred with knockdown of CTGF in the burn wounds.

**Nanoemulsions** Nanoemulsions are thermodynamically stable carrier systems of small size, with a low polydispersity index and high kinetic stability that are formed spontaneously by water, oil and surfactants. Nanoemulsions formed by oils with antimicrobial properties (e.g. *Cleome viscosa* essential oil; garlic, cinnamon and clove oils) demonstrate antibacterial activities [103–105]. The oil in a nanoemulsion can physically fuse with the lipids of microbial outer membranes, leading to membrane destabilization and lysis of pathogens [106]. Nanoemulsion formulations have been shown to exhibit broad antimicrobial properties and are widely used to treat burn wounds [107,108]. For example, Thakur *et al.* employed fusidic acid-loaded cationic bilayered nanoemulsions to prevent bacterial penetration and act as a drug reservoir [109]. Cationic bilayered nanoemulsions have multiple advantages in burn wounds, such as enhanced drug permeation, reduced bacterial load, accelerated wound contraction and facilitation of re-epithelialization. Both *ex vivo* and *in vivo* studies have verified that treating burn wounds with cationic bilayered nanoemulsions can rapidly decrease bacterial counts. Furthermore, these nanoemulsions can form a continuous film over the wound surface that can improve healing. Nanoemulsions composited with cationic or nonionic surfactants are utilized to treat *P. aeruginosa* and *S. aureus* infected burn wounds [110]. Interestingly, in rats bearing burn wounds, nanoemulsions can significantly decrease colonies of both bacterial species, reduce inflammation and facilitate wound healing progression. Bromelain is known in clinical practice for debridement in burn treatment, but it is easily inactivated by light, high temperatures and high pH values [111]. To increase the efficacy and stability of bromelain in such treatment, Rachmawati *et al*. prepared a bromelain-encapsulated nanoemulsion [112]. They then evaluated its efficacy on the burnt skin of rabbits by observing wound contraction, eschar score, erythemic score, pus score and oedema. Their results verified that the nanoemulsion showed better activity than free drug. In conclusion, compared with conventional administration, nanoemulsion showed enhanced antibacterial activity that is beneficial to accelerating the healing of burn wounds.

**Figure 7.**
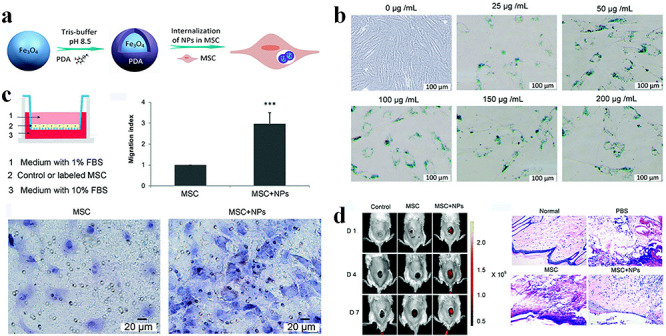
Fe_3_O_4_@polydopamine (Fe_3_O_4_@PDA) nanoparticles (NPs)-labelled mesenchymal stem cells (MSCs) for treatment of burn wounds in rats. (**a**) Fe_3_O_4_@PDA NPs preparation and internalization by MSCs. (**b**) The viability and proliferation potential of Fe_3_O_4_@PDA NPs-labelled MSCs. (**c**) Effects of Fe_3_O_4_@PDA NPs on MSCs migration *in vitro.* (**d**) Effects of MSCs on burn injury and their therapeutic effects in a living rat model. *FBS* fetal bovine serum [117]. (Copyright 2019 by Royal Society of Chemistry)

**Liposomes and solid lipid NPs** Liposomes are closed vesicles with one or more waterborne chambers formed by dispersing insoluble phospholipids and other amphiphilic substances in water. A variety of natural and synthetic phospholipids are available for the preparation of liposomes, such as phosphatidyl choline, ceramide cholesterol and phosphatidyl ethanolamine. Liposomes can directly fuse with the bacterial cell membrane and release drugs either into its interior or within the membrane. They can also be used effectively to cover wounds to create a moist environment on the wound surface, which is very conducive to wound healing [113]. GFs (e.g*.* bFGF, EGF, KGF) and photosensitizers (e.g. chlorine e6) are encapsulated into liposomes to treat various types of burn wounds. For instance, Lu *et al*. prepared trans-retinoic acid deformable liposomes and EGF cationic deformable liposomes for the treatment of deep partial-thickness burns (Figure 6a) [114]. The results of a scratch wound recovery assay suggested that both types of liposome not only synergistically enhanced cell proliferation and migration but noticeably boosted wound healing and improved healing quality when incorporated together into an ointment matrix (Figure 6b, c). Histopathological examination further confirmed that these liposomes could promote skin appendage formation and increase collagen production, thereby improving healing quality. The authors also found that *trans*-retinoic acid significantly upregulated the expression of EGFR and heparin-binding epidermal growth factor (HB-EGF) to enhance the therapeutic effect of EGF. The dual liposome ointment might serve as a promising topical therapeutic for burn wound treatment.

Solid lipid NPs-encapsulated fusidic acid has also been used to treat burn wounds, since fusidic acid can inhibit bacterial translation by blocking bacterial protein synthesis [115]. Thakur *et al.* fabricated fusidic acid-loaded lipid-polymer hybrid NPs and used these solid lipid NPs against bacteria in MRSA-infected burn wounds [116]. Lipid NPs coated with cationic chitosan can enhance the permeation and retention of fusidic acid across skin layers, which is beneficial to delivering fusidic acid into the deep dermis/epidermis milieu. Therapeutic efficacy was further assessed in a model of murine burn wounds infected with MRSA with parameters such as bacterial burden, wound contraction, and morphological and histopathological examinations of wounds. Bacterial counts decreased drastically on day 3, and wounds shrank significantly on day 5. In summary, liposomes can provide a moist environment on the surface of wounded skin because of their effective closure of epidermal cells to promote wound healing, and therefore they are widely used to treat various types of burn wounds.

In summary, polymeric nanotherapeutics have been extensively studied as treatments for various kinds of burn wounds due to their excellent biocompatibility, biodegradation and high therapeutic efficacies. In particular, biomacromolecules and drugs can be continuously released from polymeric nanotherapeutics to maintain consistent drug concentrations in wounds, which might permit dosing frequency to be decreased. In addition, the bioavailability of biomacromolecules and drugs can be improved, as NPs can be internalized by macrophages and other cells via endocytosis or pinocytosis.

**Table 4:** Assessment of burn wounds with nanotherapeutics treatment

**Assessment methods**	**Assessment items**	**Outcomes**
Counting of bacterial colonies	*E. coli, P. aeruginosa, S. aureus, P. vulgaris, C. albicans, C. freundii, K. pneumonia, C. glabrata*	Nanotherapeutics significantly reduce bacterial infection at burn wounds
Macroscopic observation	Healing rate of wounds	Nanotherapeutics obviously facilitate wound healing
Histopathology	Re-epithelialization, coagulation, vascular growth, dermis, hypodermis, panniculus carnosus, subcutaneous layer, adipose tissue, granulation tissue, regenerated sebaceous glands, skin appendage, neutrophils, scab, ulceration, thickness of epidermis, number of keratinocytes, hair roots, hair follicles	Nanotherapeutics can promote re-epithelialization, vascular growth, granulation tissue formation to boost burn wounds healing
Cytokines	Interleukins (IL-1a, IL-1β, IL-2, IL-4, IL-6, IL-8, IL-10, IL-13)	Nanotherapeutics can reduce inflammatory factors expression and increase growth factors expression to relieve inflammation and accelerate tissue formation
	Growth factors (KGF, IGF-1, IGF-BP3, FGF, TGF-β, PDGF, HB-EGF, CTGF, α-VEGF, bFGF)	
	Chemokines (CXCL1, CXCL2, CINC-1, CINC-3)	
	Tumor necrosis factor (TNF-α)	
	Transforming growth factor-β family (TGF-β)	
	Adhesion molecules (ICAM1)	
	GM-CSF	
Enzymes	Lipid peroxidation, superoxide dismutase, glutathione peroxidase, myeloperoxidase, MMP-2, MMP-9, mitochondrial respiratory chain complexes I, II, III and IV	Some nanotherapeutics exhibit anti-oxidation activity
Others	Tissue inhibitor of metalloproteinases 1 (TIMP1), α-smooth muscle actin (α-SMA), collagen type 1 alpha1 (Col1a1), C-reactive protein (CRP),	Nanotherapeutics can decrease α-SMA expression to reduce scar formation

*KGF* keratinocyte formation growth factor, *IGF* insulin-like growth factor, *IGF-BP* insulin like growth factor binding protein, *FGF* fibroblast growth factor, *TGF-β* transforming growth factor beta, *PDGF* plateletderived growth factor, *HB-EGF* heparin binding epidermal growth factor, *CTGF* connective-tissue growth factor, *VEGF* vascular endothelial growth factor, *bFGF* basic fibroblast growth factor, *GM-CSF* granulocyte macrophage colony stimulating factor, *MMP* matrix metalloproteinase

**Other nanotherapeutics** In addition to the aforementioned nanoformulations, other innovative strategies have also been developed to treat burn wounds. For example, mesenchymal stem cell (MSC)-based therapy is a promising strategy for tissue regeneration and repair. To enhance migration of MSCs to wound tissues, Li *et al.* prepared Fe_3_O_4_@polydopamine (Fe_3_O_4_@PDA) NPs-labelled MSCs and evaluated their effect at the injury site (Figure 7a) [117]. *In vitro* cell assays indicated that MSCs labelled with Fe_3_O_4_@PDA NPs did not affect cell proliferation (Figure 7b). By contrast, Fe_3_O_4_@PDA NPs could enhance the migratory ability of the MSCs by upregulating the expression levels of chemokine receptors (Figure 7c). The researchers intravenously administrated Fe_3_O_4_@PDA NPslabelled MSCs to rats with burns and performed live imaging to monitor MSCs migration. The *in vivo* images showed that Fe_3_O_4_@PDA NPslabelled MSCs had prolonged retention time at burn injury lesions (Figure 7d). Importantly, the group of Fe_3_O_4_@PDA NPslabelled MSCs-injected rats showed less inflammation than rats injected with unlabelled MSCs. Ag NPs-loaded dendrimer or TiO_2_/Ag-encapsulated poly(caprolactone) (PCL) NPs have also been used to treat burn wounds in mouse models [118,119].

### Assessment of burn wounds treated with nanotherapeutics

Both *in vitro* and *in vivo* models have been extensively employed to evaluate the therapeutic efficacies of nanotherapeutics. Anti-bacterial, anti-inflammatory and cell proliferation experiments have been mainly used to assess *in vitro* therapeutic efficacies of nanotherapeutics. However, many studies have focused on the animal models to evaluate the *in vivo* therapeutic efficacies of nanotherapeutics. Animals such as mice [54], rats [120], rabbits [100,112], dogs [121], and piglets [93] bearing burn wounds with or without bacterial infection have been used in a broad range of studies to evaluate the therapeutic effects of nanotherapeutics on such wounds. Abazari *et al.* have synthetically reviewed the establishment of various types of burn wounds (*e.g.* second vs*.* third-degree, partial- vs*.* full-thickness) in animals [25]. The clinical application of nanotherapeutics was also studied in humans with 15–40% partial-thickness thermal burns [55]. Antibacterials, anti-inflammatories and mediation of GF expression are still the primary strategies in nanotherapeutic treatment of burn wounds. Macroscopic photography provides visual images to evaluate healing of burn wounds by nanotherapeutic treatment. Macroscopic images demonstrate that nanotherapeutics can facilitate better burn wound healing than their conventional counterparts. Bacterial colonies in wounds are also counted to assess nanotherapeutic antibacterial efficacy. Antibacterial tests indicate that nanotherapeutics can significantly reduce bacteria count and prevent infection to accelerate wound healing. Histopathological images of skin tissue are useful in investigating the mechanism of wound healing (Table 4). For example, these images can demonstrate how nanotherapeutics promote re-epithelialization, neovascularization and granulation tissue formation in wounded tissues to boost wound healing. The therapeutic mechanisms of nanotherapeutics are further revealed via detection of inflammatory cytokines and GF expression at burn wound sites (Table 4). For instance, inflammatory cytokines such as TNF-α, IL-1, and IL-6 are typically used to appraise the anti-inflammatory efficacy of nanotherapeutics. GFs including VEGF, TGF-α, TGF-β, FGF and PDGF are also detected to estimate cell proliferation after nanotherapeutics treatment. In summary, various methods have been utilized to investigate the therapeutic efficacy and mechanisms of nanotherapeutics in burn wounds.

## Conclusions

A wide range of NPs have been explored for management of burn wounds. Using nanotherapeutics to treat these wounds has some advantages, such as increasing antibacterial effect, overcoming bacterial drug resistance, facilitating cell proliferation and decreasing drug administration frequency. The therapeutic efficacy of nanotherapeutics has been evaluated in various animal models on different types and degrees of burns. Some nanotherapeutics exhibit satisfactory therapeutic effects in patients with burn injuries, making them promising candidates for further studies of their role in the management of these wounds. Although gratifying therapeutic effects have been achieved, the toxicity of nanotherapeutics due to their particular physicochemical properties cannot be ignored. How to prepare multifunctional nanotherapeutics with good biocompatibility and efficacy for the treatment of burns needs further investigation. In particular, the systemic toxicity of nanotherapeutics should be investigated in various animal models before proceeding to patient applications. How to prepare nanotherapeutics for clinical practice on a large scale must also be considered. Although some problems must be overcome before this can happen, we believe that more burn patients can profit from nanotherapeutics in the future.

## Abbreviations

AgSD: Silver sulfadiazine; α-SMA: Alpha-smooth muscle actin; BAC: Benzalkonium chloride; bFGF: Basic fibroblast growth factor; C: Collagen; CINC: Cytokine-induced neutrophil chemoattractant; Col1a1: Collagen type 1 alpha 1; CPC: Cetylpyridinium chloride; CXCL-1: Chemokine (C-X-C motif) ligand 1 protein; CRP: C-reactive protein; CTAB: Cetyltrimethyl ammonium bromide; CTGF: Connective-tissue growth factor; DMPC: Dimyristoyl-*sn*-glycero-phosphatidylcholine; DNA: Deoxyribonucleic acid; DOTAP: 1,2-Dioleoyl-3-trimethylamonium propane chloride; EC: Endothelial cell; ECM: Extracellular matrix; EGF: Epidermal growth factor; EGF CDLs: EGF cationic deformable liposomes; FBS: Fetal bovine serun; FDA: Food and Drug Administration; FGF: Fibroblast growth factor; FN: Fibronectin; GFs: Growth factors; GM-CSF: Granulocyte macrophage colony stimulating factor; HA: Hyaluronic acid; HaCaT: Human immortal keratinocyte cell line; HB-EGF: Heparin binding epidermal growth factor; hFBs: Normal human fibroblast; HM-PA: Heparin-mimetic peptide; HUVECs: Human umbilical vein endothelial cells; ICAM-1: Intercellular adhesion molecule-1; IGF: Insulin-like growth factor; IGF-BP: Insulin like growth factor binding protein; IL: Interleukin; KGF: Keratinocyte formation growth factor; MMP: Matrix metalloproteinase; MRSA, Methicillin-resistant *Staphylococcus aureus*; MSC: Mesenchymal stem cell; NGF: Nerve growth factor; NPs: Nanoparticles; PBS: Phosphate buffer saline; PC: Phosphatidyl choline; PCL: Poly(caprolactone); PDA: Polydopamine; PEG: Polyethylene glycol; PEO: Polyethylene oxide; PDGF: Platelet-derived growth factor; PSIS: Porcine-derived small intestinal submucosa; PLA: Poly(lactic acid); PLGA: Poly(lactic-co-glycolic acid); PVA: Poly(vinyl alcohol); ROS: Reactive oxygen species; SG: Silver nanoparticle gel; SF: Nanosilver foam; siRNA: Short interfering ribonucleic acid; TGF-β: Transforming growth factor beta; TIMP-1: Tissue inhibitor of metalloproteinase-1; TNF-α: Tumor necrosis factor alpha; TRA: All-*trans* retinoic acid; TRA DLs: All-*trans* retinoic acid loaded deformable liposomes; TSP: Thrombin-sensitive protein; VEGF, Vascular endothelial growth factor.

## Funding

This work supported by the Open Project Program of the State Key Laboratory of Trauma, Burn and Combined Injury, Third Military Medical University (No. SKLKF201905, SKLKF201918).

## Authors’ contributions

R.H. drafted the manuscript. J. H. and D. Z. designed this project and revised the manuscript. W. Q. prepared the revised manuscript. L. C. prepared some figures and tables. All authors read and approved the final manuscript.

## Conflict of interest

The authors declared that they have no conflicts of interest to this work.
